# Final 2 year results of the vascular imaging of acute stroke for identifying predictors of clinical outcome and recurrent ischemic eveNts (VISION) study

**DOI:** 10.1186/1471-2261-11-18

**Published:** 2011-04-23

**Authors:** Shelagh B Coutts, Michael D Hill, Misha Eliasziw, Karyn Fischer, Andrew M Demchuk

**Affiliations:** 1 Room C1261, Calgary Stroke Program, Department of Clinical Neurosciences and Radiology, University of Calgary, 1403 29th St NW, Calgary, AB, T2N 2T9, Canada; 2 Calgary Stroke Program, Department of Clinical Neurosciences, Radiology, Community Health Sciences, and Medicine, University of Calgary, 1403 29th St NW, Calgary, AB, T2N 2T9, Canada; 3 Department of Community Health Sciences, University of Calgary, 1403 29th St NW, Calgary, AB, T2N 2T9, Canada; 4 Calgary Stroke Program, Department of Clinical Neurosciences, University of Calgary, 1403 29th St NW, Calgary, AB, T2N 2T9, Canada; 5 Calgary Stroke Program, Department of Clinical Neurosciences and Radiology, University of Calgary, 1403 29th St NW, Calgary, AB, T2N 2T9, Canada

**Keywords:** Magnetic resonance Imaging, Stroke, outcome, recurrent stroke

## Abstract

Among patients with ischemic stroke, little attention has been paid to differentiation between stroke progression and recurrence. We assessed the role of MR imaging in predicting stroke progression, recurrent stroke, and death within 2 years of symptom onset.

**Methods:**

Ischemic stroke or TIA patients were prospectively enrolled. They were examined within 12 hours and had a stroke MR completed within 24 hours of symptom onset. Patients were closely followed neurologically and examined if there was any deterioration in neurological status. Relationships between baseline clinical and imaging factors and outcomes were assessed. We also examined whether baseline stroke/TIA severity (NIHSS 0-5 versus NIHSS > 5) modified these relationships.

**Results:**

A total of 334 patients were enrolled. The overall rates of progression, 2-year recurrence, and 2-year death were 8.7%, 8.0%, and 6.6%, respectively. Event rates were similar among patients with mild compared to more severe strokes: 8.3% versus 9.5% (p = 0.73) for progression, and 7.3% versus 9.9% (p = 0.59) for recurrence. The effect of baseline glucose > 8 mmol/l was consistent in predicting stroke progression, recurrent stroke and death, regardless of baseline stroke severity. In multivariable analyses, DWI lesion and intracranial occlusion predicted stroke progression only in the minor stroke/TIA group; symptomatic Internal Carotid Artery (ICA) stenosis predicted stroke recurrence only in the minor stroke/TIA group.

**Conclusions:**

In a prospective study with early assessment and imaging we have found that stroke progression is different than stroke recurrence. Different imaging factors predict stroke progression versus stroke recurrence. Baseline hyperglycemia, a potentially modifiable factor, consistently predicted all three outcomes (stroke progression, recurrent stroke or death) regardless of baseline stroke severity.

## Background

Predicting outcome after an acute ischemic stroke is an important [1,2] both for treatment decisions and for discussion of prognosis with the patient and their family. Even though stroke progression and stroke recurrence are important for prognosticating patient outcome, they are not often differentiated from each other because this early outcome has not been assessed by careful neurological observation during the first 48 hours.

Prediction of outcome after stroke in the longer term includes functional outcome, identification of stroke recurrence or progression, and risk stratification [3]. Various clinical factors including age, [4-6] symptom severity as assessed by the baseline NIHSS [7] and the presence of diabetes mellitus [5,8] have been shown to correlate well with short-term outcome [9]. Age and diabetes mellitus have also been shown to predict recurrent stroke in the longer term [10,11]. Both of these factors have been included in the ABCD^2 ^Transient Ischemic attack (TIA) prognostic rule to predict early recurrent stroke [12].

Imaging is also a central part of the diagnosis and treatment of acute ischemic stroke. In most parts of the world computed tomography (CT) remains the most common modality used to assess hyperacute stroke patients, including those with TIA or minor stroke. However, Magnetic resonance imaging (MRI) has been shown to be better at identifying acute and chronic ischemia than CT alone [13]. Images from CT [14,15] and MRI [16] have been shown to correlate with short-term prognosis. A number of groups have attempted to create scores that combine many of the clinical and imaging factors, to predict short-term recurrent stroke and outcome [16,17].

None of these groups have looked at the usefulness of imaging predictors of short-term stroke progression and long-term recurrent stroke. It is well accepted in the stroke community that vessel occlusion and infarction are predictive of outcome and this may be due to stroke progression [18] or recurrent stroke [19]. Whether brain parenchymal imaging is also predictive of recurrent stroke in the longer term is not well characterized. MRI may have a particular role in the assessment of some specific patient groups, such as the minor stroke and TIA population [20].

We therefore hypothesized that the usefulness of acute MRI in predicting outcome would depend on whether the event was related to stroke progression or to a distinct recurrent stroke within 2 years of symptom onset, or to death. We limited the analysis to clinical and imaging parameters available acutely in the emergency room. As well, we hypothesized that the predictive ability could be modified by the severity of the presenting stroke or TIA.

## Methods

The data described in this paper are the 2-year final results of the prospective Vascular Imaging of acute Stroke for Identifying predictors of clinical Outcome and recurrent ischemic eveNts (VISION) study. The VISION study was a single centre prospective cohort study approved by the conjoint health research ethics board at the University of Calgary. All subjects or their surrogate provided written consent to be included in the study. Inclusion criteria were ischemic stroke or TIA (TIA patients needed to have motor or speech symptoms (or both) lasting at least five minutes) examined by a Stroke Neurologist within 12 hours of symptom onset, age 18 years or older, have NCCT and MRI brain completed within 24 hours of symptom onset and no pre-morbid disability (pre-stroke historical modified Rankin Scale (mRS) 0 or 1). All patients or next of kin provided written consent. Patient demographics were recorded at the time of emergency department assessment. A subset of the minor stroke and TIA patients have been previously published assessing short term outcomes [18,20-24]. All patients received standard acute and secondary prevention treatments for stroke at the discretion of the treating physician. This routinely includes antiplatelet agents, statins, anthypertensives where appropriate and anticoagulation for atrial fibrillation. Where a decision for carotid revascularisation was made patients were treated as soon as possible with an aim for this to be completed within 2 weeks if symptom onset in most patients.

### Imaging

Patients were not enrolled until after the baseline CT so that non-ischemic stroke diagnoses were excluded. All patients then underwent multimodal MRI of the brain and intra/extracranial vasculature (the ability to perform extracranial MR angiography (MRA) was only possible approximately half of the way through this study) within 24 hours of symptom onset using a 3-Tesla MR scanner. To be included patients had to have completed the diffusion weighted imaging (DWI) sequence of the MRI. Sequences included sagittal T1, axial T2, axial FLAIR, DWI (B = 1000), Gradient echo (GRE), perfusion imaging and 3D time-of-flight MR angiography of the intracranial (pre- and post- gadolinium) and extracranial circulation.

All imaging was interpreted blind to clinical information other than symptom side. CT images were reviewed by a stroke neurologist and were assessed for evidence of early ischemic changes. MRI of the brain was assessed by a neuroradiologist for the presence of an acute stroke lesion. The MRA was assessed using source images and maximum intensity projection reformats for evidence of an intracranial vessel occlusion, vascular occlusion or stenosis, previous strokes, microbleeds (these were defined as small regions of hypointense signal on GRE or on the first 12 T2* PW Images obtained before gadolinium contrast arrival) and leukoaraiosis (this was defined as the presence of any T2 or FLAIR white matter lesion not related to the acute infarct). Perfusion imaging was assessed for the presence of an abnormal area of perfusion. The mean transit time (MTT) map was compared with the DWI and assessment of the relative sizes of the MTT map and the area of restricted diffusion was carried out. If MTT > DWI then this was rated as "mismatch" [21]. An overall assessment of the presence or absence of symptomatic extracranial carotid artery disease (≥ 50% stenosis) was made by a stroke neurologist who combined all imaging and clinical data.

### Clinical assessment

Clinical measurements completed included NIHSS at baseline, 24 hours, and 90 days. All patients were admitted to hospital and treated by a stroke neurologist who monitored their course in hospital. If a patient deteriorated neurologically (defined as neurological worsening that affected function, persisting for at least 24 hours that was not caused by metabolic, infection or other such factors), the patient was either classified as a recurrent stroke or as stroke progression [18]. There was not a strict time threshold for the diagnosis of progression, but a temporal association with the index event was required. For example, initial mild hemiparesis evolving to hemiplegia over the first 24 hours was considered stroke progression rather than recurrent stroke, unless imaging clearly showed a distinct second event remote from the presenting event. New symptoms in a new vascular territory were considered recurrent stroke. This included assessment of the clinical and imaging information by two stroke neurologists independent of the 90 day assessment and each event was classified. The area of abnormal perfusion and follow up imaging was used to help classify events. All patients who deteriorated had follow up imaging with either CT or MRI.

The modified Questionnaire for verifying stroke free status (QVSFS) [25] was completed by telephone at 6, 12, 18 and 24 months by either a physician or a registered nurse who were blind to clinical information. If a patient scored positive on the QVSFS for a stroke then a stroke neurologist confirmed the diagnosis and the date of recurrent stroke was recorded. If the patient died during the follow up period the date of death was also recorded.

### Statistical Analyses

Only variables available at first presentation in the emergency room were considered in the analysis. We first assessed the relationship between each baseline characteristic and the outcomes of progression, recurrence, and death by calculating hazard ratios (and two-sided 95% confidence intervals) from univariate Cox proportional hazards regressions. For the purpose of analyses, patients were categorized into two groups, those with mild stroke/TIA (NIHSS 0 to 5) and those with more severe stroke/TIA (NIHSS > 5). As we hypothesized a priori that stroke severity (defined by NIHSS) could have a modifying effect on outcome, we included this factor both as a main effect and interaction term in all subsequent regression models. Given the large number of baseline characteristics and relatively few outcome events, we strategically selected the remaining variables for inclusion in the final multivariable regression model. Only variables that had p-values < 0.05 from the univariate analysis, after adjusting for multiple testing using the False Discovery Rate correction procedure [26] and p-values < 0.10 from the interaction with the dichotomized NIHSS score were considered in the model. A p-value < 0.10 was chosen because it is known that tests of interaction are underpowered [27]. All analyses were adjusted for glucose level, as this was the only factor that was consistently and significantly related to all three outcomes. The proportional hazards assumption was assessed and found to be valid for all models. Kaplan-Meier cumulative failure curves were plotted to illustrate the trajectories of progression, recurrence, and death. Cumulative predicted failure curves from the Cox regressions were plotted for recurrence by baseline characteristics. The prognostic accuracies of the models were quantified by areas under the curve (AUC) from receiver operating characteristic (ROC) analyses. Statistical analyses were performed using SAS 9.2 software (SAS Institute Inc).

## Results

A total of 334 patients were prospectively enrolled. The median age was 70 years (range: 18-95 years). The median time from symptom onset to arrival in the Emergency Department was 1.9 hours (range: 0-11.4 hours) and the median time to MRI was 7.25 hours (range: 1.3-23.4 hours). Patients were followed for a median of 23.9 months from symptom onset. Among the 334 patients, 229 (68.6%) were in the mild stroke/TIA category (including 95 classical definition TIA's [28]) and 105 (31.4%) in the moderate/severe category. During the course of the 2-year study, 29 patients had stroke progression, 23 had recurrent strokes, and 20 died. Three patients who had stroke progression died and 6 patients with recurrent stroke died.

**Figure 1:**
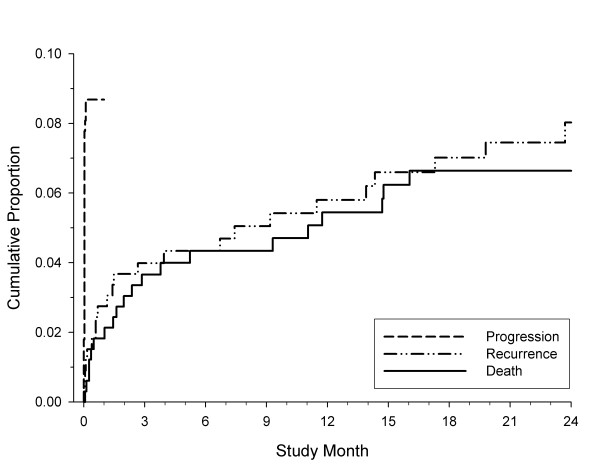
**Kaplan-Meier cumulative failure curves for the outcome of progression, recurrence and death**.

Figure 1 shows the overall cumulative failure curves for progression, recurrent stroke and death. It is apparent that progression occurred early, all within the first three days of symptom onset, and at a rate of 8.7%. In contrast, stroke recurrence and death occurred more gradually, at approximately a linear rate, with 2-year risks of 8.0% and 6.6%, respectively. For both stroke progression and stroke recurrence, the event rates were similar among patients with mild stroke/TIA compared to more severe strokes: 8.3% versus 9.5% (p-value = 0.73) for progression, and 7.3% versus 9.9% (p-value = 0.59) for recurrence. However, patients with more severe TIA/strokes had a higher death rate than those with mild TIA/strokes, 16.8% versus 1.9% (p-value < 0.001).

Univariate analysis of the clinical and imaging factors and outcomes of progression, recurrent stroke, and death are shown in Table 1. Different factors are relevant predictors of the three different outcomes. DWI lesion, intracranial occlusion and symptomatic ICA stenosis predicted stroke progression. Male sex, history of diabetes mellitus, microbleeds, and symptomatic ICA stenosis predicted recurrent strokes. NIHSS, age, and microbleeds predicted death. Most notably, admission hyperglycemia was the only factor that was consistently and highly predictive of all three outcomes, and it was unmodified by stroke severity (p-value for tests of interaction = 0.56, 0.40, and 0.40 for progression, recurrence, and death, respectively).

**Table 1:** Baseline Clinical and Imaging Characteristics and Univariate Analysis from Cox Proportional Hazards Regression

	**Percentage of Group (N = 334)**	**Progression Hazard Ratio (95% CI)**	**P-value (FDR)**	**Recurrence Hazard Ratio (95% CI)**	**P-value (FDR)**	**Death Hazard Ratio (95% CI)**	**P-value (FDR)**
	
NIHSS > 5	31.4	1.1 (0.5, 2.4)	0.74	1.3 (0.5, 3.0)	0.59	9.3 (3.1, 27.8)	< 0.001
Age > 75 years	34.4	0.8 (0.4, 1.9)	0.68 (0.86)	1.6 (0.7, 3.7)	0.26 (0.58)	8.3 (2.8, 24.8)	< 0.001 (0.001)
Male sex	58.1	1.0 (0.5, 2.2)	0.91 (0.95)	3.4 (1.1, 9.9)	0.026 (0.07)	0.8 (0.3, 2.1)	0.72 (0.77)
Hypertension history	60.0	1.1 (0.5, 2.3)	0.80 (0.86)	1.9 (0.8, 4.9)	0.16 (0.31)	1.3 (0.5, 3.1)	0.63 (0.77)
Diabetes mellitus history	15.0	1.9 (0.8, 4.4)	0.14 (0.23)	2.6 (1.1, 6.3)	0.035 (0.12)	1.9 (0.7, 5.3)	0.20 (0.42)
Coronary artery disease history	12.9	0.0 (---, ---)	0.98 (1.00)	0.6 (0.1, 2.6)	0.52 (0.79)	1.2 (0.3, 4.0)	0.79 (0.77)
Systolic BP > 140 or diastolic BP > 90	69.5	2.1 (0.8, 5.6)	0.12 (0.22)	1.3 (0.5, 3.2)	0.62 (0.79)	2.5 (0.7, 8.7)	0.14 (0.30)
Glucose > 8 mmol/L	18.0	2.5 (1.2, 5.4)	0.019 (0.06)	3.9 (1.7, 8.8)	0.001 (0.014)	3.2 (1.3, 7.7)	0.012 (0.06)
Treated with thrombolytic therapy	21.3	0.4 (0.1, 1.4)	0.15 (0.23)	1.0 (0.4, 2.8)	0.94 (0.95)	2.4 (1.0, 6.0)	0.051 (0.13)
Acute stroke on NCCT	34.1	1.4 (0.7, 2.9)	0.39 (0.53)	0.8 (0.3, 2.1)	0.71 (0.81)	1.2 (0.5, 3.1)	0.62 (0.77)
DWI lesion (one or more vs none)	68.0	6.5 (1.5, 27.3)	0.011 (0.013	2.3 (0.8, 6.8)	0.13 (0.30)	1.9 (0.6, 5.7)	0.25 (0.44)
Intracranial occlusion	21.6	5.4 (2.6, 11.3)	< 0.001 (< 0.001)	0.8 (0.3, 2.3)	0.69 (0.79)	1.6 (0.6, 4.2)	0.32 (0.57)
DWI/MTT mismatch	21.3	2.0 (0.9, 4.3)	0.075 (0.17)	1.1 (0.4, 2.9)	0.90 (0.95)	1.3 (0.5, 3.5)	0.65 (0.77)
White matter disease on MRI	70.0	1.1 (0.5, 2.6)	0.75 (0.86)	2.0 (0.7, 6.0)	0.19 (0.31)	3.9 (0.9, 16.9)	0.067 (0.13)
Microbleeds	23.6	1.7 (0.8, 3.7)	0.17 (0.23)	2.2 (1.0, 5.1)	0.061 (0.21)	2.9 (1.2, 6.8)	0.021 (0.10)
Symptomatic ICA stenosis	15.3	3.1 (1.4, 6.6)	0.004 (0.013)	3.3 (1.4, 7.7)	0.007 (0.05)	1.5 (0.5, 4.4)	0.48 (0.77)

FDR = False Discovery Rate p-value adjusting for multiple testing

Test of interaction with dichotomized NIHSS score p-value: Progression (DWI lesion = 0.10, Intracranial occlusion = 0.011); Recurrence (Symptomatic ICA stenosis = 0.05); Death (Age = 0.049)

Table 2 shows the multivariable analysis for stroke progression, recurrent stroke and death within 2 years, stratified by stroke severity. In predicting progression, stroke severity modified the relationship with DWI lesion and intracranial occlusion. Both factors were only predictive of stroke progression among patients with mild strokes/TIAs (p-value for tests of interaction = 0.13 and 0.022, respectively) but not in patients with NIHSS > 5. The two imaging factors increased the AUC prognostic accuracy from 0.59 (with only NIHSS and glucose in the model) to 0.79 when imaging was included.

**Table 2:** Final Multivariable Cox Proportional Hazards Regression Models for Progression, Recurrence, and Death

	**NIHSS 0 to 5 (N = 229)**		**NIHSS > 5 (N = 105)**	
	**Hazard Ratio (95% CI)**	**P-value (FDR)**	**Hazard Ratio (95% CI)**	**P-value (FDR)**
	
**Progression (19 and 10 events)**				
DWI lesion (one or more vs none)	12.4 (1.6, 93.3)	0.014 (0.028)	1.4 (0.2, 11.1)	0.75 (0.82)
Intracranial occlusion	11.3 (4.4, 29.2)	< 0.001 (< 0.001)	1.9 (0.5, 6.5)	0.33 (0.53)
**Recurrence (15 and 8 events)**				
Symptomatic ICA stenosis	5.6 (2.0, 15.6)	< 0.001 (0.004)	0.6 (0.1, 4.9)	0.64 (0.82)
**Death (4 and 16 events)**				
Age > 75 years	1.3 (0.1, 13.7)	0.82 (0.82)	19.5 (2.6, 148.3)	0.004 (0.011)

Each analysis was adjusted for glucose

FDR = False Discovery Rate p-value adjusting for multiple testing

In predicting recurrent stroke, stroke severity modified the relationship with symptomatic ICA stenosis. The single factor was predictive of recurrent stroke in patients with mild strokes/TIAs (p-value for tests of interaction = 0.058), but not in patients with NIHSS > 5. The single imaging factor increased the AUC prognostic accuracy from 0.64 (with only NIHSS and glucose in the model) to 0.77 when imaging was included. No imaging factors were predictive of death. Only older age predicted death, and only for patients with severe (NIHSS > 5) strokes/TIAs (p-value for tests of interaction = 0.062). Age did not significantly increase the prognostic accuracy for death (AUC of 0.87 versus 0.80 for NIHSS and glucose alone).

**Figure 2:**
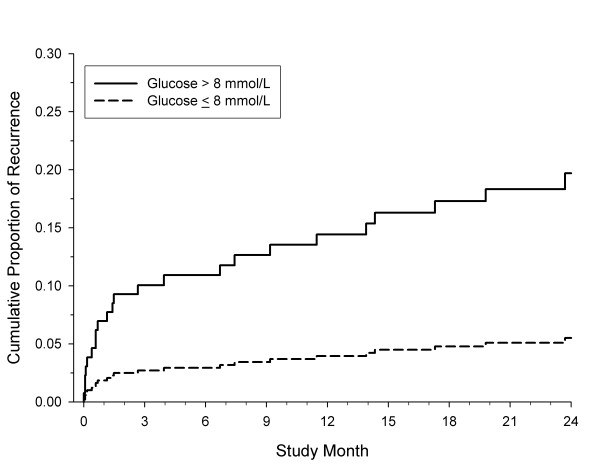
**Cumulative predicted failure curves from Cox regression for the outcome of recurrence by baseline glucose level**.

**Figure 3:**
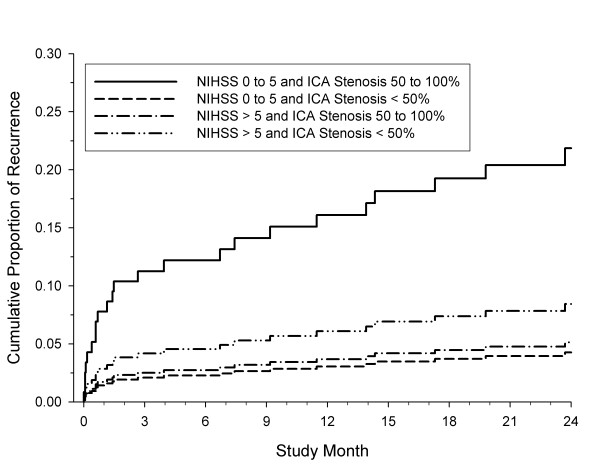
**Cumulative predicted failure curves from Cox regression for the outcome of recurrence by baseline NIHSS score and degree of symptomatic ICA stenosis, adjusting for baseline glucose level**.

Figure 2 and 3 show the cumulative predicted failure curves from Cox regression for the outcome of recurrence by baseline glucose level and by degree of symptomatic ICA stenosis, respectively. Admission hyperglycemia has a detrimental effect on stroke recurrence, regardless of stroke severity. However, stroke recurrence was higher only among patients with a mild stroke/TIA who had moderate-to-severe ICA stenosis (50 to 100%).

## Discussion

The clinical and imaging factors that predict stroke progression and recurrent stroke at 2 years are different. We have, with careful neurological assessment, distinguished between recurrent stroke and stroke progression. Baseline hyperglycemia was important for all outcomes of stroke progression, recurrent stroke and death within 2 years in both groups of patients. We found that the overall rates of stroke progression and recurrent stroke were similar between the TIA/minor stroke and the moderate/severe groups, although there were differences in the predictors between the 2 groups.

Different imaging parameters are useful at different times. Stroke progression is an acute phenomenon occurring most commonly in the first 24 hours. The presence of a DWI lesion and/or intracranial vascular occlusion predicted stroke progression in the minor stroke/TIA population. This argues for acute MR imaging of these patients in the first 24 hours. Among patients with an intracranial occlusion symptom progression may be due to failure of collaterals or less commonly to recurrent embolus. In the minor stroke/TIA population the addition of the imaging parameters described above, to the clinical factors increases the AUC substantially for predicting both stroke progression and recurrent stroke. This suggests that early MRI should be the modality of choice for TIA and minor stroke. Assessing the imaging predictors of recurrent stroke we find that carotid stenosis is most likely a reflection of the underlying risk factors for vascular disease. Carotid stenosis is well understood as a strong predictor of long term stroke risk [29].

All patients in this protocol-driven study were enrolled by stroke neurologists, underwent detailed clinical and imaging evaluations, and were followed prospectively. The advantage of this is that we were able to distinguish between stroke progression and recurrent stroke. Stroke progression and recurrent stroke appear to be different in mechanism as well as timing [30]. Progression happens extremely early and is related to the presenting event (which is why imaging early matters); however, recurrence very early is less common and is more relevant as a longer term event. Over 2 years, recurrence is related to risk factors for vascular disease. Interestingly we found a similar stroke progression and recurrent stroke rate between the minor stroke/TIA and the moderate/severe stroke populations.

Our results are consistent with the previous literature that has shown that long-term prognosis after stroke is more dependent on the underlying vascular risk factors (including stroke mechanism) than factors related to the presenting event [31-34]. This has not been previously shown for hyperacute MRI. Additional advantages of having an MRI completed early, such as confirmation of ischemia, identifying the vascular territory affected and assessment of the final etiology of the stroke or TIA [35], all may alter management, suggest to us that MR should be the modality of choice of the TIA/minor stroke population.

We have shown the importance of hyperglycemia on the prediction of stroke progression, recurrent stroke and death within 2 years. Hyperglycemia may be the result of an acute stress response or be associated with previously undiagnosed or latent diabetes mellitus. In our view, the relationship with recurrent stroke at 2 years makes the latter more likely in this population. Hyperglycemia is an important poor prognostic factor in acute stroke [36] and it remains an important area for further study. Recent preliminary trials of aggressively targeting normoglycemia have been negative or neutral, but this is an important area for further study as it is a potentially modifiable factor [37].

There are some limitations to this work. This is a single centre observational study and these results need replicated in other centres. The cause of death was not captured in this study and so we cannot assess how many patients died from a vascular death. Some groups have advocated the use of DWI volumes to predict outcome after stroke, [38] but there have been mixed results in the literature with regards this [39]. We chose only to assess factors available on initial assessment in the emergency room and as DWI volumes are not routinely measured in clinical practice we did not include this in our analysis. Another limitation is that the ability to image the neck vessels with MR angiography was only available approximately half way through the study, so assessment of neck vessels was made using various combinations of imaging modalities; including MR angiography, CT angiography and Carotid Doppler. Although this is a limitation it is the reality of non invasive imaging. Also the relatively smaller number of patients in the moderate/severe group compared to the minor stroke/TIA group is one possible explanation for not finding MRI parameters that predict outcome in this subgroup.

## Conclusions

In a prospective study we have found that the adding imaging results to clinical parameters increases the accuracy of predicting recurrent stroke and stroke progression. We also found that the imaging features that were important differed for recurrent stroke and stroke progression. This work is hypothesis generating and needs replicated in other centres.

## Competing interests

The authors declare that they have no competing interests.

## Authors' contributions

SC conceived the study, participated in the study design and coordination and drafted the manuscript. MH participated in the study design, helped with the statistical analysis and helped to draft the manuscript. ME participated in the design of the study and performed the statistical analysis. AMD conceived the study, participated in its design and coordination and helped to draft the manuscript. KF participated in the study design and coordination and edited the manuscript. All authors read and approved the final manuscript.

## Pre-publication history

The pre-publication history for this paper can be accessed here:

http://www.biomedcentral.com/1471-2261/11/18/prepub
